# Auditory stimulation with music influences the geometric indices of heart rate variability in men

**DOI:** 10.1186/1755-7682-7-27

**Published:** 2014-05-22

**Authors:** Sheila Ap F da Silva, Heraldo L Guida, Ana M dos SantosAntônio, Luiz Carlos M Vanderlei, Lucas L Ferreira, Luiz Carlos de Abreu, Fernando H Sousa, Vitor E Valenti

**Affiliations:** 1 Centro de Estudos do Sistema Nervoso Autônomo (CESNA), Departamento de Fonoaudiologia, Faculdade de Filosofia e Ciências, Universidade Estadual Paulista, UNESP, Av. Hygino Muzzi Filho, 737, 17525-900 Marília, SP, Brasil; 2 Programa de Pós-Graduação em Fisioterapia, Faculdade de Ciências e Tecnologia, Universidade Estadual Paulista (UNESP), Rua Roberto Simonsen, 305, 19060-900 Presidente Prudente, SP, Brasil; 3 Departamento de Morfologia e Fisiologia, Faculdade de Medicina do ABC, Av. Príncipe de Gales, 821, 09060-650 Santo André, SP, Brasil; 4 Faculdade de Filosofia, Ciências e Letras de Ribeirão Preto, Universidade de São Paulo, USP, Av. Bandeirantes, 3900, 14040-90 Ribeirão Preto, SP, Brasil

**Keywords:** Autonomic nervous system, Auditory stimulation, Cardiovascular system, Music

## Abstract

**Background:**

Chronic classical music was reported to increase parasympathetic activitywhen evaluating heart rate variability (HRV). It is poor in the literature investigation of the acute effects of baroque and heavy metal styles of musical auditory stimulation on HRV. In this study we evaluated the acute effects of relaxant baroque and excitatory heavy metal music on the geometric indices of HRV in healthy men.

**Method:**

The study was performed in 12 healthy men between 18 and 30 years old. We excluded persons with previous experience with music instrument and those who had affinity with the song styles. We analyzed the following indices: RRtri, TINN and Poincaré plot (SD1, SD2 and SD1/SD2 ratio). HRV was recorded at rest for ten minutes. Subsequently they were exposed to relaxant baroque or excitatory heavy metal music for five minutes through an earphone. After the first music exposure they remained at rest for more five minutes and them they were exposed again to Baroque or Heavy Metal music (65–80 dB). The sequence of songs was randomized for each individual.

**Results:**

The RRTri and SD2 indices were reduced during the heavy metal musical auditory stimulation (p < 0.05). No changes were observed regarding TINN, SD1 and SD1/SD2 ratio (p > 0.05).The qualitative Poincaré plot analysis indicated that during relaxant classical baroque music there was observed a higher beat-to-beat dispersion of RR intervals compared with no music exposure and during excitatory heavy metal musical auditory stimulation, showing higher HRV.

**Conclusion:**

We suggest that excitatory heavy metal music acutely decreases global HRV.

## Background

Therapy with music has been investigated to treat cardiac dysfunction [1]. For instance, a recent study [1] indicated that music help in improving sleep quality of subjects with acute and chronic sleep disorders. Also, a recent review published by our group [2] presented some studies that showed the chronic benefic effects of relaxant music on the cardiovascular system [3-6]. A previous and very interesting study performed by Bernardi and colleagues [7] studied 24 healthy young adults. The authors investigated the effects of orchestra, music with vocals and progressive crescendos on heart rate, respiratory rate, blood pressure and middle cerebral artery flow. It was also noted reduced activity of the sympathetic nervous system.

Conversely, heavy metal music was indicated to cause negative effects, which were related to stress responses. It is suggested that heavy metal music increases sympathetic activity whereas classical music reduces the sympathetic nervous system activity and increase parasympathetic activity [8].

In this context, the heart rate variability (HRV) is a non-invasive method for investigation of autonomic nervous system (ANS). This method analyzes the oscillations of the intervals between consecutive heartbeats. This method is a conventionally accepted term to describe the fluctuations in the intervals between consecutive heartbeats (RR intervals), which are indicated to influence the sinusal node [9].

Among the methods used for HRV analysis we may include the geometric methods, which is composed by the Poincaré plot - which convert RR intervals into geometric patterns and allow analyzing HRV through the geometric or graphic properties of the resulting pattern, triangular interpolation of NN interval histogram (TINN) and the triangular index (RRtri) [9]. Moreover, some authors consider the Poincaré plot analysis as based on nonlinear dynamics [9].

It was reported the benefic effects of musical auditory stimulation for long time [5]. Great part of studies investigating the cardiovascular responses induced by musical auditory stimulation used stereo-system tools to exposure the subjects or animals [4,5]. A previous study showed that the intensity of the auditory brainstem responses to rising chirp stimulation is influenced by the amplitude-frequency response induced by the earphone [10]. Additionally, another study indicated that white noise exposure through earphone influence the cardiac autonomic activity [11]. However, it is not clear if the use of earphones cause different responses of the HRV.

Therefore, this investigation was undertaken to analyze the acute effects of excitatory heavy metal and relaxant classical baroque musical auditory stimulation on HRV geometric indices in healthy men.

## Method

### Study population

We analyzed 12 male healthy subjects aged between 18 and 30 years old, selected from our Institution.All volunteers were informed about the procedures and objectives of the study and, after agreeing, have signed a term of informed consent. All study procedures were approved by the Ethics Committee in Research of the Faculty of Sciences of the Universidade Estadual Paulista, Campus of Marilia (Case No. CEP-2011-382) and followed the resolution 196/96 National Health 10/10/1996.

### Non-inclusion criteria

We did not include subjects under the following conditions: audiological and cardiopulmonary disorders, neurological and other impairments that prevent the subject known to perform procedures, and treatment with drugs that influence cardiac autonomic regulation. We did not include subjects with previous experience with music instrument and classic ballet music, as well as volunteers which like heavy metal and classical music styles, since it affects cardiovascular responses [12].

### Initial evaluation

Before the experimental procedure, volunteers were identified by collecting the following information: age, gender, weight, height and body mass index (BMI). Anthropometric measurements were obtained according to Lohman et al. [13]. Weight was determined by using a digital scale (W 200/5, Welmy, Brazil) with a precision of 0.1 kg. Height was determined by using a stadiometer (ES 2020, Sanny, Brazil) with a precision of 0.1 cm and 2.20 m of extension. Body mass index (BMI) was calculated using the following formula: weight (kg)/height (m)^2^.

### Measurement of the auditory stimulation

The measurements of the equivalent sound levels were conducted in a soundproof room, using a SV 102 audiodosimeter (Svantek, Poland). It was programmed the measurement in the "A" weighting circuit; slow response.

The measurement was made during a session, lasting a total of four minutes and 50 seconds for the relaxant classical baroque music and five minutes and 15 seconds for the excitatory heavy metal music. We used the insert type microphone (MIRE - Microphone in real ear), which was placed inside the auditory canal of the subject, just below the microphone, connected to the personal stereo.

Before each measurement, the microphones were calibrated with the calibrator acoustic CR: 514 model (Cirrus Research plc).

We used in the analysis was the Leq (A), which is defined as the equivalent sound pressure level and corresponds to the constant sound level in the same time interval. It contains the same total energy of the sound, we also analyzed the frequency spectrum of the sound stimulation (octave band). The equivalent sound level was between 64-85 dB.

### HRV analysis

The R-R intervals recorded by the portable HR monitor (with a sampling rate of 1000 Hz) were downloaded to the Polar Precision Performance program (v. 3.0, Polar Electro, Finland). The software enabled the visualization of HR and the extraction of a cardiac period (R-R interval) file in "txt" format. Following digital filtering complemented with manual filtering for the elimination of premature ectopic beats and artifacts, at least 256 R–R intervals were used for the data analysis. Only series with more than 95% sinus rhythm was included in the study [9,14]. For calculation of the indices we used the HRV Analysis software (Kubios HRV v.1.1 for Windows, Biomedical Signal Analysis Group, Department of Applied Physics, University of Kuopio, Finland).

### Geometric indices of heart rate variability

HRV analysis was performed by means of geometrical methods: RRtri, TINN and Poincaré plot (SD1, SD2 and SD1/SD2 ratio). The RRtri was calculated from the construction of a density histogram of RR intervals, which contains the horizontal axis of all possible values of RR intervals measured on a discrete scale with 7.8125 ms boxes (1/128 seconds) and on the vertical axis, the frequency with which each occurred. The union of points of the histogram columns forms a shape like a triangle. The RRtri was obtained by dividing the total number of RR intervals used to construct the histogram by their modal frequency (RR interval value that most frequently appeared on RR) [14].

The TINN consists of the measure of the base of a triangle. The method of least squares is used to determine the triangle. The RRtri and the TINN express the overall variability of RR intervals [9].

The Poincaré plot is a map of points in Cartesian coordinates, constructed from the values of RR intervals obtained, where each point is represented on axis x (horizontal/abscissa) by the previous normal RR interval, and on axis y (vertical/coordinate), by the following RR interval.

For quantitative analysis of the plot, an ellipse was fitted to the points of the chart, with the center determined by the average RR intervals, and the SD1 indexes were calculated to measure the standard deviation of the distances of the points to the diagonal y = x, and SD2 measures the standard deviation of the distances of points to the line y = - x + RRm, where RRm is the average of RR intervals. The SD1 is an index of instantaneous recording of the variability of beat-to-beat and represents parasympathetic activity, while the index SD2 represents HRV in long-term records, and reflects the overall variability. Their ratio (SD1/SD2) shows the ratio between short and long variations of RR intervals [9].

The qualitative analysis of the plot was made through the analysis of the figures formed by its attractor, which were described by Tulppo et al. [15] in:

Figure in which an increase in the dispersion of RR intervals is observed with increased intervals, characteristic of a normal plot.

Small figurewith beat-to-beat global dispersion without increased dispersion of RR intervals in the long term. We used the software HRV analysis.

### Experimental protocol

Data were collected in a room with the temperature set between 21°C and 25°C and relative humidity regulated between 50% and 60%. The volunteers were instructed not to drink alcoholic or caffeinated beverages for 24 hours before the evaluation. The data were collected on an individual basis between 8 AM and 12 PM. The procedures necessary for the data collection were explained on an individual basis; the subjects were instructed to remain at rest and avoid talking during the data collection.

After the initial evaluation, we placed the heart monitor belt over the subject's thorax, aligned with the distal third of the sternum, and the Polar RS800CX heart rate receiver (Polar Electro, Finland) was placed on the wrist. The subjects were seated and remained at rest with spontaneous breathing for 10 minutes with the earphones turned off.

The protocol was performed on the following sequence: 1) Record of 10 minutes with no music exposure; 2) After 10 minutes of rest, the subjects were exposed to excitatory heavy metal (Gamma Ray's "Heavy Metal Universe") or classic baroque (Pachelbel's "Canon in D Major") musical auditory stimulation for 5 minutes each style; 3) subsequently, the individuals remained at rest for 5 minutes and; 4) thereafter they were exposed to musical auditory stimulation for 5 minutes. The sequence of songs was randomized for each individual [16,17].

### Statistical analysis

In order to calculate sample size we applied the power analysis that provided a minimal number of 10 subjects. Standard statistical methods were used for the calculation of means and standard deviations. Normal Gaussian distribution of the data was verified by the Shapiro-Wilk goodness-of-fit test (z value >1.0). For parametric distributions we applied the one way ANOVA for repeated measures followed by the posttest of Bonferroni (SD1 index). For non-parametric distributions we used the Friedman test followed by the Dunn’s posttest (TINN, RRTri and SD2 indices and SD1/SD2 ratio). We compared the geometric indices of HRV between the three moments (rest vs. 1^st^ music vs. 2^nd^ music). Differences were considered significant when the probability of a Type I error was less than 5% (p < 0.05). We used the Software GraphPad StatMate version 2.00 for Windows, GraphPad Software, San Diego California USA.

## Results

Table 1 presents the values regarding basal diastolic (DAP) and systolic arterial pressure (SAP), heart rate (HR), mean RR, weight, height and body mass index (BMI) of the volunteers.

**Table 1:** Mean ± standard-deviation of baseline diastolic (DAP) and systolic arterial pressure (SAP), heart rate (HR), mean RR interval, weight, height and body mass index (BMI) of the volunteers

**Variable**	**Value**
Age (years)	21.7 ± 3
Height (m)	1.74 ± 0.09
Weight (kg)	62 ± 10
BMI (kg/m^2^)	21.3 ± 3
HR (bpm)	76 ± 7
Mean RR (ms)	739.1 ± 87
SAP (mmHg)	112 ± 9
DAP (mmHg)	64 ± 13

Legend: m: meters; ms: millisecond; kg: kilograms; bpm: beats per minute; mmHg: millimeters of mercury.

Table 2 indicates that the SD1 index during exposure to excitatory heavy metal music style tended to be reduced compared to the control group, but showed no statistical significant changes compared to control. The same occurred with the index TINN, which tended to be decreased during excitatory heavy metal music exposure with no significant difference compared to control. The RRtri showed a significant reduction during exposure to the same style of musical auditory stimulation compared to control. Moreover, the SD2 index showed significant reduction during both excitatory heavy metal music compared to control condition. Regarding the SD1/SD2 ratio, it almost reached statistically significant reduced values during heavy metal music exposure compared to control.

**Table 2:** Mean ± Standard deviation (minimum-maximum) of the analysis of HRV in geometric indexes in comparison with the control group and Baroque music with heavy metal music

**Index**	**Control**	**Recovery 1**	**Baroque music**	**Recovery 2**	**Heavy metal music**	**p**
**RRTri**	13.3 ± 5 (4.2-23.1)	13.1 ± 4 (4–21.3)	13.5 ± 3 (5.7-16.2)	13.2 ± 3 (4.1-20.4)	11.3 ± 3 (5.67-17.2)*	0.04
**TINN**	235.4 ± 83 (65–365)	231.2 ± 80 (61–355)	241.7 ± 61 (85–305)	233.1 ± 81 (62–361)	202.1 ± 56 (75–260)	0.07
**SD1**	21.2 ± 9 (4.8-31.4)	20.3 ± 8 (4.3-30.3)	21.2 ± 8 (6.1-33.3)	20.9 ± 9 (4.1-29.2)	20.5 ± 10 (5.5-36.1)	0.09
**SD2**	69.4 ± 21 (21–97.7)	69.3 ± 19 (20–99.8)	70.3 ± 17 (27–85.9)	68.1 ± 21 (19–98.9)	59 ± 15 (24.7-82.4)*	0.03
**SD1/SD2**	0.29 ± 0.11 (0.11-0.53)	0.28 ± 0.12 (0.11-0.52)	0.28 ± 0.16 (0.01-0.68)	0.29 ± 0.1 (0.12-0.51)	0.34 ± 0.18 (0.14-0.82)	0.076

Legend: RRtri – Triangular index; TINN – triangular interpolation of RR intervals; SD1 – standard deviation of the instantaneous variability of the beat-to beat heart rate; SD2 – standard deviation of long-term continuous RR interval variability; SD1/SD2 ratio – ratio between the short - and long - term variations of RR intervals.

*p < 0.05: vs. Control

**Figure 1:**
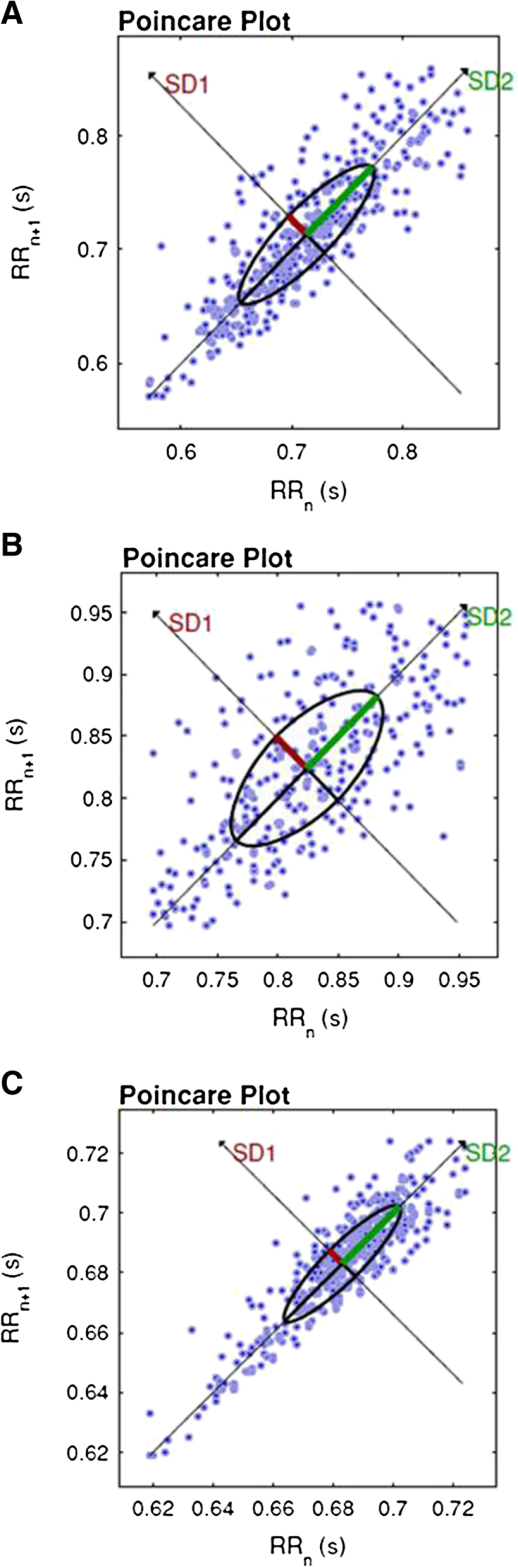
Visual pattern of Poincaré plot observed in one subject during control condition (A), exposure to relaxant baroque music (B) and excitatory heavy metal music (C).

Figure 1 shows an example of Poincaré plot patterns from one subject during no music (A), relaxant baroque (B) and excitatory heavy metal musical auditory stimulation (C).

## Discussion

As a main finding, we reported that excitatory heavy metal musical auditory stimulation acutely affects the global HRV while no significant change was observed during exposure to relaxant classical baroque music in men.

We observed that the SD2 index was significantly reduced during excitatory heavy metal musical auditory stimulation compared to control. The SD2 index corresponds to HRV in long-term records, it represents the global variability of heart rate [9], indicating that this style of music reduced HRV. Two studies performed by Chuang et al. [3,6] showed that the SDNN, the time-domain index that corresponds to the global HRV is positively influenced by relaxant music. On the other hand, the effect of heavy metal music on global HRV was not investigated yet.The literature indicated that some styles of music, such as techno music, hip hop and heavy metal [18] are usually related to arousal responses, but not pathological [19]. A previous investigation observed that musical auditory stimulation with techno style induced increases in cortisol, norepinephrine, and adrenocorticotropic hormone levels related to the sympathetic nervous system and heart rate [20,21]. As evidence, our data indicated that this style of music acutely reduced HRV in men, suggesting acute decrease of cardiac autonomic regulation during this situation.

According to our findings, the RRTri was also decreased in response to excitatory heavy metal musical auditory stimulation. This index corresponds to global HRV [9]. Moreover, decreased levels of global HRV are related to increased risk for sudden cardiac death in subjects with coronary artery disease and it is also related to several mortality causes [22]. Based on our results and according to the literature cited above, we suggest that excitatory heavy metal musical auditory stimulation acutely reduces cardiac autonomic regulation in healthy men, supporting the findings reported regarding the SD2 index.

The qualitative analysis of the Poincaré plot reported that during relaxant classical baroque musical auditory stimulation it was observed a higher beat-to-beat dispersion of RR intervals, as well as greater dispersion of RR intervals in the long term, compared with no music exposure and during excitatory heavy metal musical auditory stimulation, indicating that relaxant classical baroque music acutely increases HRV. It is important to mention that previous studies indicated the Poincaré plot analysis as based on nonlinear dynamics. Nonlinear analysis of HRV is suggested to be more sensitive in response to changes in cardiac autonomic regulation compared to the linear analysis [9]. Taken together, it is proposed that the qualitative analysis of the Poincaré plot indicates changes that are not observed in the quantitative analysis.

The literature indicated benefic chronic benefic effects of relaxant music on cardiac autonomic regulation. Anthracycline-treated breast cancer patients were treated with music therapy for ten months, the authors observed improvement of the linear indices of HRV, indicating better cardiac autonomic regulation [6]. Mozart’s Symphony No. 40 was reported to acutely reduce basal heart rate with no effect on arterial blood pressure in rats [23]. Another study showed that auditory stimulation with Chopin’s Etude did not affect renal sympathetic nerve activity and arterial blood pressure while musical auditory stimulation with Schumann’s Träumerei suppressed the same variables in urethane anesthetized rats [5]. The authors suggested that only some styles of musical auditory stimulation influence sympathetic activity and blood pressure in rats. The same group using similar protocol indicated that gastric vagal nerve activity is increased in response to musical auditory stimulation with Traeumerei by Schumann music [4]. In our study the subjects were exposed to musical auditory stimulation through earphones, while in the studies cited above the subjects and animals were exposed through stereo-system equipment. We may raise the hypothesis that relaxant musical auditory stimulation through earphone affects with less intensity the cardiac autonomic responses.

Great part of the studies investigated musical auditory stimulation through stereo-systems [4-6]. Those studies indicated that relaxant music increases the parasympathetic activity on the heart. Conversely, we found no acute effects of relaxant classical baroque musical auditory stimulation on the geometric indices of HRV. It was previously indicated that the intensity of the auditory brainstem responses to rising chirp stimulation is affected by the amplitude-frequency response of the earphone [24]. Considering that the brainstem regulates the cardiovascular system [2,25,26], it could be hypothesized that the use of earphones influences cardiovascular reaction during auditory stimulation. However, it lacks in the literature the influence of earphones on cardiovascular responses induced by auditory stimulation with music. We encourage additional studies to further investigate this mechanism.

According to our results, the SD2 index was reduced during excitatory heavy metal musical auditory stimulation through earphones. A recent study indicated that rotating musical stimulation, a type of relaxant auditory stimulation, influenced the SD2 [27]. The authors observed that this type of auditory stimulation increased SD2. The rotating stimulation proposed by the authors was based on an Indian percussion instrument table. The stimulation was digitally recorded at a sampling rate of 44.1 kHz in 16 bit. Taken together, we may argue that this post stimulation reduction in the global variability of heart rate, measured by SD2, is not a favorable result of auditory stimulation with excitatory heavy metal music.

In our study the volunteers were exposed to an auditory stimulation between 64-85 dB during the relaxant classical baroque music protocol and between 75-84 dB during excitatory heavy metal music. Lee and coworkers [11] investigated the effects of white noise in different equivalent sound levels on HRV. White noise is a random signal with a flat (constant) power spectral density that present no change in the equivalent sound level [28]. The authors reported no significant effect on basal heart rate and arterial blood pressure. The LF index was increased in response to five minutes of white noise in a low to moderate intensity, ranging from 50 to 80 dBA. It was also observed strong correlation between noise intensity (dBA) and the LF index. On the other hand, there was no change in the parasympathetic activity of the heart during exposure to different intensity of white noise. It is hard to verify the effects of different equivalent sound levels of music because each music presents a different range of this variable, as we observed in the "Canon in D" composed by Pachelbel compared with the "Heavy Metal Universe" music composed by Gamma Ray.

Some mechanisms are proposed to explain the responses observed in the geometric indices of HRV during excitatory heavy metal musical auditory stimulation. A previous study performed in rats [4] indicated that not all types of music cause the same effect, as we reported in our study. The authors reported that a style of relaxant music induce cardiovascular responses and changes in the autonomic nervous system through histaminergic neurons in the suprachiasmatic nucleus of the hypothalamus, which depends on an intact cochleae, on the auditory cortex and on the primary somatosensory cortex. Another study showed that the pleasure induced by musical auditory stimulation may cause dopamine release in the nucleus accumbens [29]. In this context, the nucleus accumbens is a striatal area involved in the reward system.

Our study presents some points that are worth to be raised. Based on our results, we are not able to conclude the the long-term effect of musical auditory stimulation on HRV. A small population was studied, however, statistical analysis provided difference. This issue deserves further investigation to support this mechanism. We investigated only men, these results can not be extrapolated to women. We decided to study one single gender because there is difference between men and women regarding music-induced physiological responses. It is suggested a tendency for women to present increased stress reactive compared with men [19]. The authors reported that during excitatory heavy metal musical auditory stimulation women presented more intense increase in the sympathetic responses compared to men [19]. We investigated a small population, however, the statistical analysis provided significance for the indices.

## Conclusion

We reported that acute excitatory heavy metal musical auditory stimulation decreased the geometric indices of HRV, while no changes were observed during relaxant classical baroque musical auditory stimulation. There was no significant change during Classic Baroque musical auditory stimulation compared with the control group. We suggest that acute auditory stimulation with excitatory heavy metal music decreases the cardiac autonomic regulation. Future studies are necessary to support this result.

## Competing interests

The authors declare that they have no competing interests.

## Authors’ contributions

All authors participated in the acquisition of data and revision of the manuscript. All authors determined the design, interpreted the data and drafted the manuscript. All authors read and gave final approval for the version submitted for publication.
